# Accuracy of genotype imputation of a low-density SNP array for the Amazon fish *Colossoma macropomum*

**DOI:** 10.1590/1678-4685-GMB-2023-0364

**Published:** 2024-09-02

**Authors:** John F. G. Agudelo, Vito A. Mastrochirico-Filho, Baltasar F. Garcia, Raquel B. Ariede, José M. Yáñez, Gustavo M. R. Valladão, Diogo T. Hashimoto

**Affiliations:** 1 Universidade Estadual Paulista (Unesp), Centro de Aquicultura da Unesp, Jaboticabal, SP, Brazil.; 2 Universidade Estadual Paulista (Unesp), Faculdade de Ciências, Bauru, SP, Brazil.; 3 Universidad de Chile, Facultad de Ciencias Veterinarias y Pecuarias, Santiago, Chile.; 4 Universidade Nilton Lins, Manaus, AM, Brazil.

**Keywords:** Tambaqui, SNP Array, genotyping, breeding programs, genetic improvement

## Abstract

In South America, Tambaqui (*Colossoma macropomum*) stands as the primary target for aquaculture, yet breeding programs for this Amazon native species are in their early stages. While high-density single nucleotide polymorphism (SNP) arrays are pivotal for aquaculture breeding, their costs can be prohibitive for non- or semi-industrial species. To overcome this, a cost-effective approach involves developing low-density SNP arrays followed by genotype imputation to higher densities. In this study, a 1K SNP array for tambaqui was created and validated, offering a balance between SNP quantity and genome representativity. The imputation accuracy from various SNP densities to a medium-density array was evaluated, with the 1K density demonstrating the best trade-off (accuracy of 0.93). This subset was further utilized to construct a commercial array through Agriseq™ targeted genotyping-by-sequencing, validated in 192 DNA samples, affirming its high quality for genotyping tambaqui. The low-density SNP array, with genome-wide coverage and high polymorphism, emerges as an effective tool for exploring genetic variation within diverse populations. Population analyses using the 1K panel proved to be an efficient tool for genetic characterization of sampled broodstocks, making it a valuable resource for genetic improvement programs targeting this Amazon native species.

## Introduction

The tambaqui fish (*Colossoma macropomum*) is classified as an omnivorous species, with preference for frugivorous behavior, in contrast to its closely related predatory counterparts, the piranhas of the Serrasalmidae family (Woynárovich and Van Anrooy, 2019). This unique species exhibits remarkable resilience, rapid growth, artificial feed acceptance, substantial productivity, and holds significant commercial value in international markets (Valladão et al., 2018). Tambaqui stands out as the principal Amazon fish species cultivated in Brazil, particularly concentrated in the northern region of the country (IBGE, 2022). As evidenced by statistics from the Brazilian Institute of Geography and Statistics (IBGE, 2022), aquaculture production of tambaqui attained 94.6 thousand tons in 2021. Moreover, this remarkable fish species possesses desirable characteristics that make it advantageous to aquaculture development in Latin America, such as adaptability to different environments, high feed conversion efficiency and market demand (Hilsdorf et al., 2022). As a result, tambaqui aquaculture has experienced successful expansion beyond Brazil, extending to countries such as Bolivia, Colombia, Peru, Ecuador, and Venezuela (Valladão *et al*., 2018).

In Brazil, most of the native fish aquaculture relies on unselected stocks, in which breeding programs are still in the beginning stage of development. Genetic selection programs have been initiated in tambaqui targeting efficient growth and disease resistance (Agudelo et al., 2022). As research and breeding techniques continue to advance, the application of genomic selection for complex traits is poised to revolutionize tambaqui aquaculture and contribute significantly to the industry’s growth. This modern approach allows fish breeders to make more precise breeding decisions, thereby accelerating the genetic progress of tambaqui populations. One of the primary challenges in implementing genomic selection-based breeding programs for native species is associated with the high cost of investment. In addition, a significant portion of native fish production is carried out by small to medium-scale producers, who generally lack the financial solvency and specialized workforce to invest in genetic improvement programs.

Recently, more comprehensive genomic studies have been conducted on tambaqui, including the sequencing of a reference genome (Hilsdorf et al., 2021), population genetic studies (Agudelo et al., 2022), linkage mapping, identification of Quantitative Trait Loci (QTLs) (Ariede *et al*., 2020, 2022), and the development of a dense array of Single Nucleotide Polymorphisms (SNPs) (Mastrochirico-Filho et al., 2021). However, the high cost of genotyping thousands of SNPs for accurate genomic prediction in a species primarily utilized in small-scale aquaculture, remains a limiting factor in the routine application of genomic selection programs.

An alternative approach to mitigate costs involves the adoption of lower density marker panels and the imputation of unobserved genotypes using data from higher density SNP arrays of related animals, thereby enhancing the genomic prediction accuracy (Phocas, 2022). Recent research has assessed the imputation accuracy from low- to high density panels in different fish species such as, *Salmo salar* (Tsai et al., 2017; Yoshida et al., 2018; Tsairidou et al., 2020; Kriaridou et al., 2023) and *Oreochromis niloticus* (Garcia et al., 2022). Nevertheless, a fully validated low-density SNP array suitable for genotype imputation at a commercial scale is yet to be made available in tambaqui.

A low-density SNP panel combined with imputation techniques may be used to decrease costs of genotyping without compromising the reliability of genomic predictions. This study evaluated the feasibility of genotype imputation from five different low SNP densities (0.5, 1, 2, 3 and 9K) using as reference a medium-density 30K SNP array (Mastrochirico-Filho et al., 2021). We used an experimental tambaqui breeding population from Brazil to validate the results and offer a cost-effective solution for genotyping without compromising accuracy.

## Material and Methods

### Ethics statement

This study was conducted in strict accordance with the recommendations of the National Council for Control of Animal Experimentation (CONCEA) (Brazilian Ministry for Science, Technology and Innovation) and was approved by the Ethics Committee on Animal Use (CEUA number 1038/21) of Faculdade de Ciências Agrárias e Veterinárias, UNESP, Campus Jaboticabal, SP, Brazil.

### Experimental population and SNP genotyping

The population under investigation in this study constitutes the breeding population maintained at the Aquaculture Center, São Paulo State University (UNESP), located in Jaboticabal city, São Paulo State, Brazil. The animals used for SNP genotyping were previously described in an analysis of GWAS for resistance against *Aeromonas hydrophila* in tambaqui by Ariede *et al*. (2020, 2022), which consisted of 18 half and full-sib families. Breeding involved 9 females and 14 males, with four males each mating with two females (resulting in four half-sibling groups, equivalent to eight full-sibling families), three males each mating with one female (forming one half-sibling group, equivalent to three full-sibling families), two males each mating with one female (yielding one half-sibling group, equivalent to two full-sibling families), and five males and five females paired individually (resulting in five full-sibling families). To obtain these families, induced spawning was conducted using carp pituitary extract (Danubio piscicultura LTDA), administered in two doses with a 12-hour interval. The first and second dosages were 0.5 and 5.5 mg/kg, respectively, and a single dosage of 2.5 mg/kg of carp pituitary extract was used for males, simultaneously with the second dosage for females (Pinheiro-Lima and Silva, 1988).

Genomic DNA was extracted from blood samples obtained from 272 fish, consisting of 34 parents and 238 offspring, using the commercial Wizard Genomic Kit from Promega, following the manufacturer’s instructions. The quantity and quality of the DNA were evaluated in the following steps. Firstly, a NanoDrop spectrophotometer^®^ from Thermo Fisher Scientific was utilized, which allowed the quantification of DNA to check concentration and purity. Secondly, agarose gel electrophoresis was performed to further verify the integrity and quality of the DNA samples. To quantify the extracted DNA accurately, the Qubit fluorescence detector, in conjunction with the Qubit dsDNA BR Assay kit^®^ from Invitrogen, was employed. This approach enabled precise measurements of DNA concentration in units of nanograms per microliter (ng/μl). The application of these techniques ensured a comprehensive assessment of the DNA’s quantity and quality, facilitating reliable subsequent analyses and experiments.

SNP genotyping of the fish was conducted using the multispecies SerraSNP Affymetrix^®^ Axiom^®^ array, a custom-designed array developed by our research group and commercially available for pacu *Piaractus mesopotamicus* and tambaqui (Mastrochirico-Filho et al., 2021). The SerraSNP Affymetrix^®^ Axiom^®^ array comprises 29,575 SNPs specific for tambaqui. The genomic DNA samples were sent to Thermo Fisher Scientific, located in California, USA, for the genotyping process. The raw data, containing the intensity calculation results (CEL files), was imported into the Axiom Analysis Suite (v2.0.035, Affymetrix) for quality control analysis and genotype calling, utilizing default parameters. To pass the initial quality control assessment, samples with a dish quality control (DQC) value exceeding 0.82 and a QC (quality control) call rate exceeding 0.97 (following the “Best Practices Workflow” recommended by Affymetrix) were considered satisfactory. Furthermore, additional quality control procedures were conducted using the software PLINK 1.9 (Chang et al., 2015), as follows: 1) Minor allele frequency (MAF) filtering was performed with a threshold of < 0.05, which was applied to the entire population, rather than within each family, to enhance the stringency in excluding potential genotyping errors; 2) Mendelian error analysis was conducted for each family, using the parameters -me 0.05 0.1, to identify and eliminate individuals and/or SNPs based on the Mendelian error rate.

### Selection of low-density subsets of SNPs for imputation accuracy

Different low-density SNP subsets were selected to evaluate the accuracy of genotype imputation. For this step, we used the genotypes of 81 tambaqui individuals from 5 different broodstock populations, considering 2 populations from North and 3 populations from Southeast regions of Brazil. All samples were genotyped by the multispecies SerraSNP Affymetrix^®^ Axiom^®^ array, according to the section 2.2. The quality control (QC) filter was carried out by the PLINK 1.9 software, using Hardy-Weinberg equilibrium (HWE) (p-value < 0.05 after Bonferroni correction), call rate of 90%, and MAF (< 0.01). In addition, the extent of linkage disequilibrium (LD) between SNPs was assessed within each broodstock population using the LD-based variant pruner by PLINK. A range of r^2^ pruning thresholds, varying from 0.9 to 0.6, was then applied to eliminate redundant combinations of SNPs. This process resulted in the selection of subsets of common SNPs among the broodstock populations, ensuring the retention of non-highly redundant genotypic information for further analyses.

### Accuracy of genotype imputation on low-density SNPs

After extracting the low-density subsets of SNPs, a cross-validation system consisting of 10 replicates was employed to evaluate the imputation accuracy in the population comprising the 34 parents and 238 offspring. In each replicate, a random subset of animals was assigned using pipelines implemented in the R Software (R Core Team, 2018). For this setup, 10% of the individuals were designated for validation purposes, while the remaining 90% served as a reference for imputation. As a result, each replicate consisted of 27 to 29 individuals for validation, leading to a total of 272 individuals used in the analysis. To simulate the imputation scenario, individuals in the validation category had their SNPs masked, leaving only the low-density subsets of SNPs representing the low-density panel. In contrast, individuals classified in the reference category remained unmodified in their genotype data. For the validation category, genotypes were imputed using the FIMPUTE3 software (Sargolzaei et al., 2014). This process was conducted across all 10 replicates to mitigate the effects of stochastic sampling. Subsequently, the imputed genotypes obtained from the validation replicates were compared to the true genotypes to determine the accuracy of the imputation. The Pearson correlation coefficient (R) was employed as a measure to assess the accuracy, and the “R” value was estimated using the following formula:

$$R=\frac{\sum_{i=1}^{n}\left(x_{i}-\overset{-}{x})(g_{i}-\overset{-}{g}\right)}{\sqrt{\sum_{i^{'}1}^{n}{\left(x_{i}-\overset{-}{x}\right)}^{2}\sum_{i^{'}1}^{n}{\left(g_{i}-\overset{-}{g}\right)}^{2}}}$$ (1)

where for each SNP in which the true genotypes were masked, the possible genotypes were coded as 0, 1, or 2, representing the potential number of the smallest allele for that SNP in the target population; *x*_*i*_ represents the imputed genotype for individual *i*, and *x* represents the mean value of the imputed genotypes across all individuals. Likewise, *ɡ*_*i*_ and *ɡ* represent the variables corresponding to the observed genotypes, and *n* denotes the number of individuals in the validation group. The calculation of R, used as a measure of imputation accuracy, was performed at both the SNP marker and individual levels. SNPs that were imputed accurately demonstrated an accuracy of over 80% (R > 0.8) across all animals. The evaluation of imputation accuracy was carried out within the chromosomes of tambaqui, which consist of 27 pairs. Considering that the ends of the chromosomes are known to have low imputation accuracy, after imputation, one hundred markers distributed at both ends of each chromosome (5,400 imputed SNPs) were evaluated in the lower density subsets (1K, and 0.5K densities).

### Development of the low-density SNP array

The development of the low-density SNP array was centered on a subset of SNPs that ensured both a reduced number of SNPs and maintained a high imputation accuracy. For constructing the array, the AgriSeq^®^ Targeted Genotyping by Sequencing (Agriseq tGBS) genotyping platform was chosen. The sequences containing the SNPs were required to be at least 400 base pairs (bp) long. All the SNPs used in the low-density array were derived from the Affymetrix^®^ Axiom^®^ SerraSNP array (Mastrochirico-Filho et al., 2021), where the SNPs are represented by 71-mer nucleotide sequences. To align these 71-mer sequences against the tambaqui genome (NCBI RefSeq assembly accession: GCA_904425465.1, assembled at the chromosome level by Ariede et al., 2022), the BLASTn pipeline was employed. The goal was to obtain the necessary total 400 bp loci for each SNP to implement the Agriseq tGBS strategy. Strict criteria were applied during the alignment process, mandating a high percentage of sequence similarity and full coverage of the query sequence concerning the genomic region of the tambaqui genome. The parameters applied were -perc_identity ranging from 97% to 99% and -qcov_hsp_perc 100%. The resulting 400 bp flanking sequences containing the SNPs were extracted using bioinformatics pipelines and subsequently submitted to Thermo Fisher Scientific for quality control analysis and primers design. 

The evaluation of the Agriseq tGBS low-density array involved genotyping a total of 192 DNA samples from tambaqui individuals for genetic population analysis, considering 96 individuals from fish farms located in the Northern region of Brazil (Amazon region), and 96 individuals produced in the Southeast region of Brazil. These 192 fish were not genotyped using the SerraSNP Affymetrix^®^ Axiom^®^ array to compare the concordance results with Agriseq tGBS. However, our research group previously documented different population structures between the North and South populations of farmed tambaqui in Brazil using SNPs from the SerraSNP Affymetrix^®^ Axiom^®^ array (Mastrochirico-Filho et al., 2021; Agudelo et al., 2022). Therefore, this result can then be validated using the Agriseq tGBS array. DNA extraction and quality parameters were performed according to the section 2.2. The genomic DNA samples were sent to Thermo Fisher Scientific in Austin, Texas, USA, for SNP genotyping. The SNP data were subjected to a QC check using the PLINK, applying the parameters MAF > 0.01, call-rate for samples and SNPs > 0.15, Hardy-Weinberg equilibrium (p = 1e-06). The performance of the developed low-density SNP array was evaluated applying the PLINK 1.9 software (Chang et al., 2015) for estimation of the MAF frequencies and mean of heterozygosity per genotyped individual. To evaluate the genetic structure among the analyzed populations, the presence of genotypic clusters among the populations from the North and Southeast regions was investigated. This was achieved by performing a two-dimensional multidimensional scaling analysis (MDS) on the genomic identity-by-state (IBS) matrix. Furthermore, population structuring analysis was performed using STRUCTURE 2.3.4 software (Pritchard et al., 2000) to estimate the optimum number of population clusters (K). The range of K values was predefined from 1 to 3. The analysis was performed in 5 replicated runs for each K value using 50,000 iterations after a burn-in period of 5,000 iterations. The determination of the number of clusters in the dataset was selecting it following the methodology described by Evanno et al. (2005) implemented in the Structure Harvester software (Earl and von Holdt, 2012). 

## Results

### Selection of low-density subsets of SNPs for imputation accuracy

**Figure 1-:**
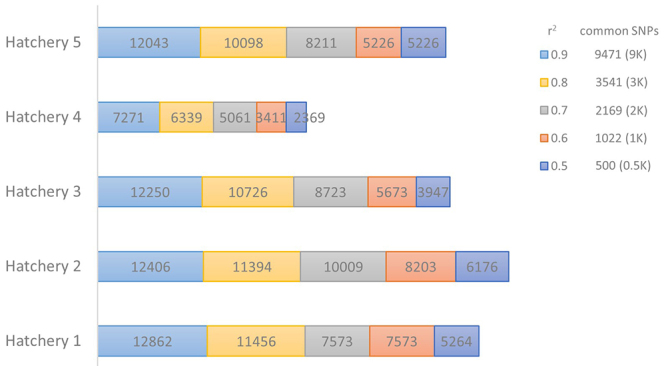
Linkage disequilibrium filtering (LD) applied to remove redundant SNP combinations. The common SNPs among the populations were used to form the low-density subsets that will be evaluated for imputation accuracy.

The QC processes to select high-quality SNPs present in each broodstock population (Broodstock 1-5), which composed the low-density subsets for the evaluation of genotype imputation accuracy, were described in Table 1 and Figure 1. The high-quality SNPs ranged from 14,985 SNPs in Broodstock 4 to 16,792 SNPs in Broodstock 5. The Broodstock 5 had a higher proportion of high-quality SNPs, with higher MAF values and genotyped markers present in most of its individuals (call rate > 0.90). On the other hand, Broodstock 4 had a higher proportion of its markers removed (approximately 13.7%) (Table 1).

**Table 1-:** Quality control (QC) process of the SNPs used to compose the low-density subsets for evaluating the accuracy of genotype imputation in tambaqui. The high-quality SNPs were filtered from five tambaqui broodstocks of fish farms.

QC process	Broodstock 1 (N = 20)	Broodstock 2 (N = 33)	Broodstock 3 (N = 9)	Broodstock 4 (N = 9)	Broodstock 5 (N = 10)
Before QC^a^	15,821	15,821	17,372	17,372	17,372
HWE^b^	15,814 (99.9 %)	15,793 (99.8 %)	17,372 (100 %)	17,372 (100 %)	17,372 (100 %)
Call rate^c^	15,814 (99.9 %)	15,793 (99.8 %)	16,682 (96.0 %)	16,710 (96.2 %)	17,296 (99.6 %)
MAF^d^	15,668 (99.0 %)	15,626 (98.8 %)	16,398 (94.4 %)	14,985 (86.3 %)	16,792 (96.7 %)

^a^ SNPs from the validation of the *Affymetrix* ^*®*^ Axiom^*®*^ SerraSNP array (Mastrochirico-Filho et al., 2021).

^b^ SNPs in Hardy-Weinberg Equilibrium (HWE) (p > 0.05, after Bonferroni correction).

^c^ SNPs with call rate > 90%

^d^ SNPs with minor allele frequency (MAF) > 0.01

In relation to the filtering of linkage disequilibrium aimed at removing redundant information among the broodstock, the squared correlation (r^2^) threshold between pairs of SNPs varied from 1 to 0.6. This process resulted in the creation of distinct low-density subsets: 9,471 SNPs (9K), 3,541 SNPs (4K), 2,169 SNPs (2K), 1,022 SNPs (1K), and 500 SNPs (0.5K) (Figure 1). The imputation accuracy of each subset was then evaluated.

### Accuracy of genotype imputation on low-density SNPs

**Figure 2 -:**
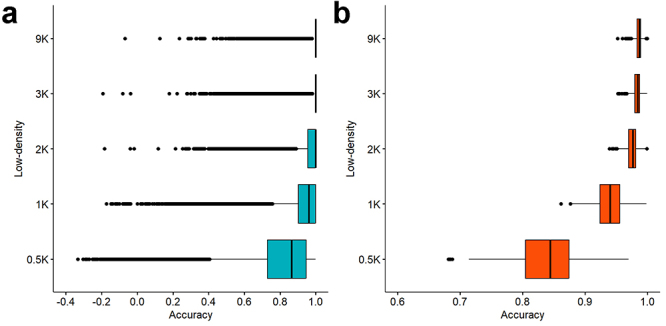
Comparing the Pearson correlation coefficient (r) between imputed and observed genotypes as a measure of imputation accuracy (R) for each low-density subsets of SNPs. The accuracy was assessed for imputed SNPs (a) and at the individual level (b).

After QC analysis, 280 genotyped samples and 17,368 SNPs were used as final dataset for assessing the accuracy of imputation. The genotype imputation accuracies were assessed both at SNP and individual levels for each density (Figure 2 and Table 2). In brief, consistently high to moderate accuracy values were maintained across densities ranging from 9K to 1K. However, a notable decrease in accuracy was observed when the density reduced to 0.5K. Specifically, at the SNP level, mean accuracies ranged from 0.98 (SD 0.04) to 0.93 (SD 0.10) for densities 9K to 1K, decreasing to 0.81 (SD 0.17) at the 0.5K density. At the individual level, the mean accuracy reached 0.83 (SD 0.05) for the 0.5K density, whereas it exceeded 0.93 for the other densities. The proportion of SNPs with accuracy greater than 0.8 was notably lower at the 0.5K density, ranging from 88.4% when assessing accuracy solely based on SNPs to 76.8% when considering accuracy based on animals. Conversely, for the other densities at the SNP level, more than 90% of the SNPs exhibited accuracies exceeding 0.8.

**Table 2 -:** The summary of the accuracy of genotype imputation using different low-density (LD) subsets of SNPs. The accuracy was described for imputed SNPs and at the individual level. The mean (SD), minimum and maximum accuracy, as well as percentage (%) of SNPs with accuracy greater than 0.8 in each scenario, were registered (% > 0.8).

		Accuracy at SNP level		Accuracy at individual level			
LD subsets	Imputed SNPs	Mean (SD)	% > 0.8	Mean (SD)	Min.	Max.	% > 0.8
9K	7,897	0.98 (0.04)	99.0	0.98 (0.01)	0.95	0.99	100
3K	13,816	0.98 (0.04)	98.9	0.98(0.01)	0.95	0.99	100
2K	14,882	0.97 (0.05)	97.9	0.98 (0.01)	0.94	0.99	100
1K	14,822	0.93 (0.10)	90.8	0.94 (0.02)	0.86	0.99	100
0.5K	16,836	0.81 (0.17)	64.0	0.84 (0.05)	0.68	0.97	76.8

**Figure 3 -:**
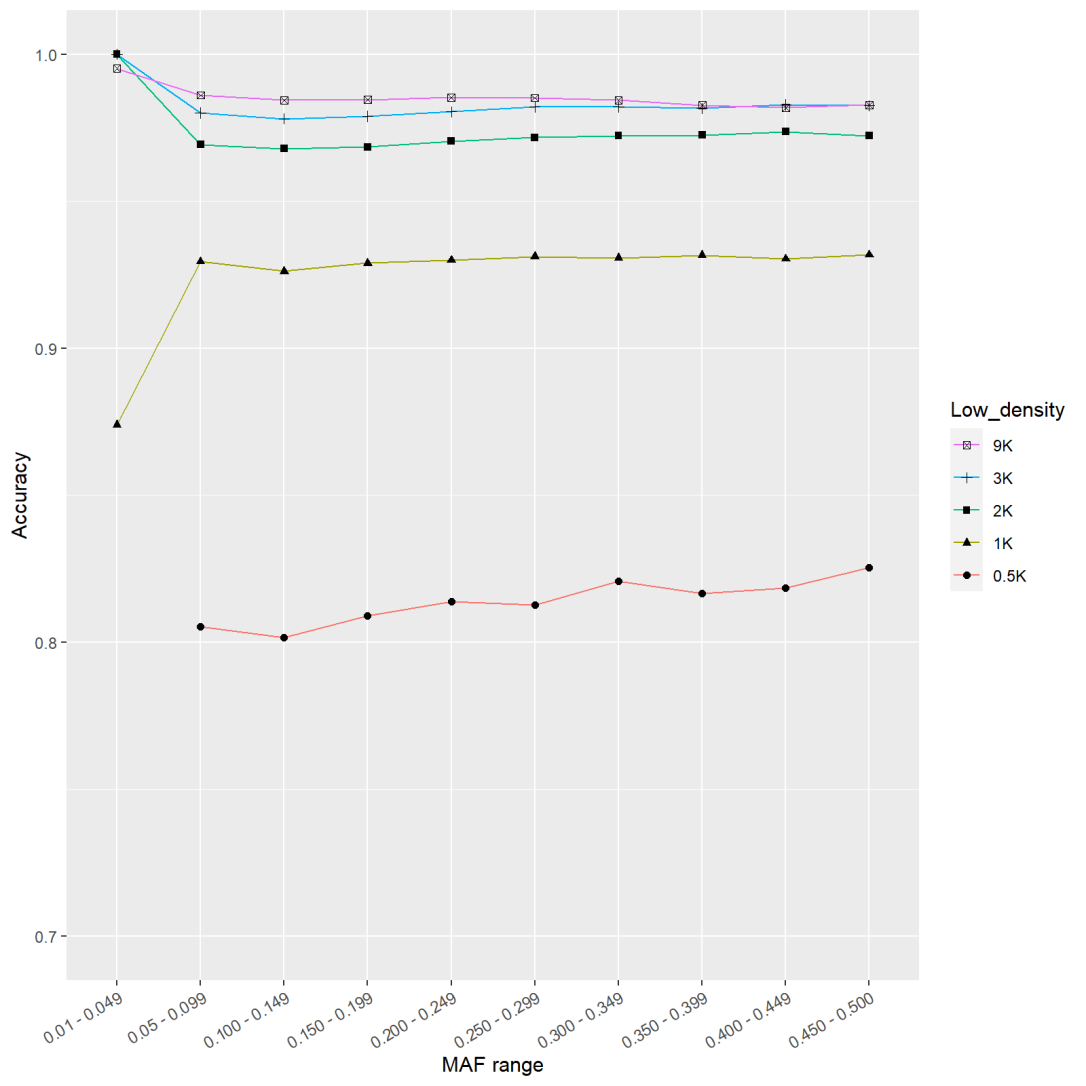
Correlation between accuracy (R^2^) and minor allele frequency (MAF) range examined for different low-density subsets: 9,471 SNPs (9K), 3,541 SNPs (4K), 2,169 SNPs (2K), 1,022 SNPs (1K), and 500 SNPs (0.5K).

The analysis of accuracy means was also conducted concerning the MAF values (Figure 3). The outcomes revealed that imputation accuracies displayed only a marginal increment in their values with the increase in MAF values. Notably, rare SNPs were absent from the analysis due to their removal during the QC steps. Interestingly, SNPs with low MAF values (0.01-0.049) exhibited high to moderate mean accuracy, surpassing even SNPs with more frequent alleles when lower densities were taken into consideration.

**Figure 4 -:**
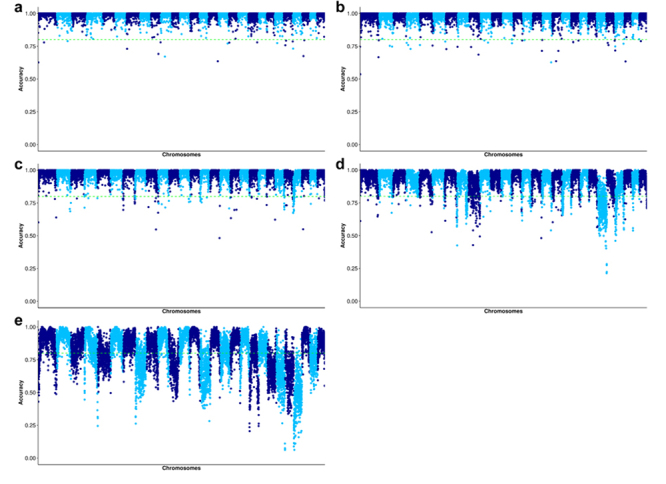
Squared Pearson correlation measure of imputation accuracy (R^2^) across the 27 chromosomes of tambaqui (*Colossoma macropomum*) after imputation using low-density subsets, included a) 9K, b) 3K, c) 2K, d) 1K, e) 0.5K SNPs.

The accuracy of imputed SNPs for each low-density subset was evaluated across the 27 chromosomes (Figures 4a-e). The findings revealed that imputed SNPs situated at both the beginning and end of each chromosome displayed lower imputation accuracy compared to SNPs located in other regions of the chromosomes. This decrease in accuracy at both ends was particularly pronounced when evaluating the 1K and 0.5K subsets (Figure S1). Nevertheless, for the 1K subset, the mean accuracy fluctuated between 0.67 (SD 0.20) and 0.96 (SD 0.07) in these regions, suggesting no significant decline in imputation accuracy (refer to Figure S1). Consequently, a substantial proportion of imputed SNPs exhibited high accuracy, enhancing the imputation performance of the 1K subset along the chromosome regions. Notably, a minority of SNPs with accuracies below 80% were more prevalent, particularly for LG22.

In contrast, the 0.5K SNP density resulted in significantly lower accuracy at the chromosome extremities, with average values ranging from 0.34 (SD 0.22) to 0.91 (SD 0.08). This indicated a higher prevalence of low and variable imputation accuracies compared to the other densities (see Figure S1). Considering the accuracy for each low-density subset, 1K SNPs were identified as the optimal density for constructing the low-density SNP array for tambaqui.

### Validation of the low-density SNP array

The SNPs amount of the 1K low-density array in the genetic map of tambaqui (Ariede et al., 2022) is succinctly outlined in Table 3. There were between 19 and 74 markers distributed across the specified chromosomal regions. This implies that approximately 3.58% to 10.33% of all SNPs covering the chromosomal regions of the genetic map were incorporated into the low-density panel.

**Table 3 -:** Characterization of the SNPs from the 1K SNP array into the genetic map of tambaqui.

Chromosomes	Genetic Map (nº SNPs)a	1K array (nº SNPs)b	% SNPsc
1	1,106	54	4.88
2	877	74	8.44
3	811	67	8.26
4	799	71	8.89
5	772	40	5.18
6	772	72	9.33
7	737	58	7.87
8	684	35	5.12
9	675	30	4.44
10	650	56	8.62
11	647	48	7.42
12	629	58	9.22
13	629	65	10.33
14	631	50	7.92
15	611	52	8.51
16	623	32	5.14
17	582	38	6.53
18	555	33	5.94
19	560	55	9.82
20	530	40	7.55
21	532	43	8.08
22	531	19	3.58
23	517	39	7.54
24	513	29	5.65
25	501	44	8.78
26	456	33	7.24
27	444	27	6.08
total	17,374	1,262	7.26

^a^ The total number of SNPs distributed in the genetic map of tambaqui (Ariede et al., 2022).

^b^ The distribution of the SNPs from the 1K SNP array into the genetic map.

^c^ Proportion (%) of the SNPs from the 1K SNP array in relation to the total of SNPs per chromosome of the genetic map.

**Figure 5 -:**
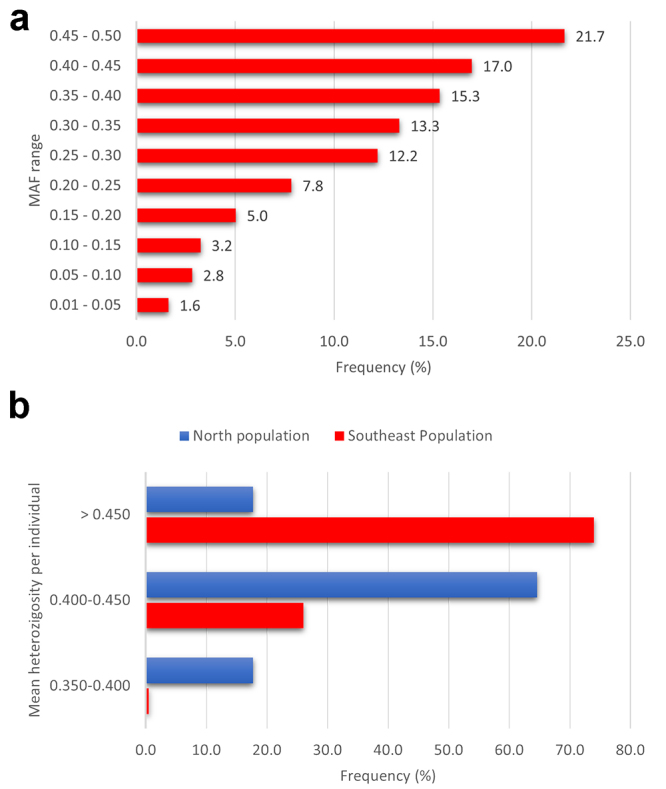
Frequency of MAF values of tambaqui populations from North and Southeast regions of Brazil (a). Mean of heterozygosity per individual from North (blue) and Southeast (red) populations (b).

For the validation of the 1K SNP array, the QC analysis of the genotyping retained 843 SNPs (70% of the total SNPs) and 169 individuals (86% of the total individuals). Low frequencies of the mutant alleles (MAF values from 0.01 to 0.05) represented only 1.6% of the total SNPs. Highly polymorphic SNPs with MAF values ranging from 0.45 to 0.50 were more abundant, representing 21.7% of the total SNPs (Figure 5a). Differences in mean heterozygosity per individual were evident, highlighting genetic distinctions between the populations in the Southeast and North regions of Brazil (Figure 5b). The Southeast population exhibited a higher frequency of heterozygous loci (74%) compared to individuals from the North population, underscoring greater genetic diversity in the Southeast.

**Figure 6-:**
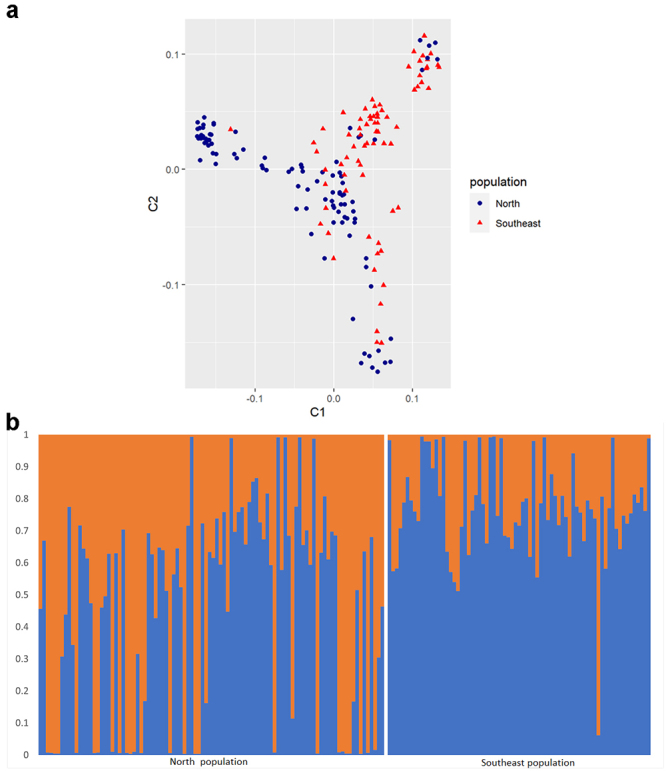
Analysis of the genetic structure of tambaqui populations from North and Southeast regions of Brazil. The figure (a) represents the multidimensional scaling (MDS) plot of the individual IBS distances for the North (blue) and Southeast (red) populations. The figure (b) represents the STRUCTURE analysis as a function of the number of putative genetic clusters (K). The genetic structure was analyzed approaching K = 2 according to the Delta K statistic. Each vertical bar represents an individual. Populations are separated by vertical white bars. The color proportions of each bar correspond to the estimated fractions of association of the individuals in each of the clusters.

The genetic structure analysis supported the hypothesis of the onset of genetic differentiation between the tambaqui populations in the Southeast and North regions (Figure 6a and Figure 6b). This validation underscores the efficacy of the low-density SNP panel in capturing genetic diversity within both populations. 

## Discussion

The developed SNP array stands as one of the pioneering low-density panels optimized through genotype imputation for the Neotropical aquaculture. This molecular tool allows substantial reduction in genotyping costs (in a scenario of genotyping 1,000 fish), from approximately $50 per sample when using the medium-density array (Axiom technology), to $10 per sample (Agriseq platform), representing a reduction of 80.0% in genotyping expenses. The low-cost genotyping strategy holds significant relevance for target species predominantly cultivated by small-scale farmers, herein exemplified by the Amazon fish tambaqui. 

Our results indicated that high imputation accuracy values (above 0.80) slightly decreased with decreasing SNP density in the validation process, a trend observed in prior studies on low-density SNP arrays for Atlantic salmon (Tsairidou et al., 2020) and Nile tilapia (Yoshida et al., 2019). Approximately 99.0% to 90.8% of genotypes achieved an accuracy above 0.80 when marker densities ranged from 9K to 1K SNPs, respectively. However, imputation accuracy sharply declined when the density decreased to 0.5K SNPs. Similar trends were noted at the genotyped animal level, with accurate imputations being more prevalent between 9K and 1K densities (100%), decreasing to 76.8% at the 0.5K density. Based on these findings, the 1K low-density genotyping array, followed by genotype imputation using as reference the 30K SerraSNP Affymetrix^®^ Axiom^®^ array, could be tested for further cost-effective genomic selection programs in tambaqui.

Previous studies have suggested a positive correlation between high MAF values and accurate imputation, in which SNPs presenting low MAF are challenging for imputation (Brøndum et al., 2012; Ma et al., 2013). Although our results revealed a subtle increase in accuracy with rising MAF values, we did not observe a high correlation between these parameters. Thus, our findings suggests that MAF values are closely linked to inaccurately imputed genotypes, and the imputation method effectively mitigated any impact of MAF on the results. Consequently, the imputation accuracy method remains preferable in imputation evaluations, as highlighted in prior studies (Calus et al., 2014; Garcia et al., 2022).

SNPs located at chromosome ends also present challenges for imputation, displaying lower accuracy across all tested densities. Accuracy notably declined, particularly with very low SNP densities, such as the 0.5K density in this study, consistent with previous research (Ventura *et al*., 2016; Yoshida et al., 2018). Chromosomal ends typically exhibit lower imputation accuracy. If traits of interest are influenced by this region, genomic association studies may introduce bias and compromise the investigation of imputed genotypes. To address this issue, including more SNPs at chromosome ends, either by increasing SNP array density or utilizing more comprehensive SNP arrays in future analyses, could serve as a potential strategy to be tested.

The genotyping technology employed for constructing the SNP array was targeted genotyping-by-sequencing (*Agriseq tGBS*), which was carefully selected for its high accuracy in genotyping heterozygous loci and lower rates of missing data (Ott et al., 2017), which was corroborated in the present study in the validation process. Moreover, the genetic diversity and structure analysis corroborated the distinct patterns previously observed between the North and South populations of farmed tambaqui in Brazil (Mastrochirico-Filho et al., 2021; Agudelo et al. 2022), and reveals the efficiency of using the 1K low-density array in population analysis.

The development of tambaqui production is currently in its early domestication stages and lacks technologies that could facilitate the establishment of effective breeding programs. The susceptibility of tambaqui stocks to diseases (bacterial and parasites), exacerbated by the intensification of production, has led to significant economic losses (Ariede *et al*., 2020; Valladão et al., 2020; Hilsdorf et al., 2022). The genetic control of disease resistance is known to be distributed among several Quantitative Trait Loci (QTLs), each elucidating only a small portion of the genetic variation. In this case, the prediction of genetic merit of animals, based on genomic estimated breeding values (GEBV, referred to as Genomic Selection), has been shown to enhance the genetic gain in aquaculture breeding (Yáñez et al., 2023). Hence, the validated strategy presented here, involving a low-density array followed by genotype imputation, can serve as a framework for efficiently incorporate disease resistance into genomic selection programs for the Amazon fish tambaqui.

## Conclusions

We proposed a cost-effective solution involving a 1K SNP array with imputation techniques, offering precise genotyping without exorbitant expenses. The 1K density, validated with high accuracy (0.93), proves to be an optimal trade-off in terms of accuracy of imputation. In addition, this genomic tool may be employed to population genomics studies in order to evaluate genetic variability and differentiation of populations.

## Supplementary material

The following online material is available for this article:

Figure S1- Mean accuracy (R2)
