# Supplementary material for: Accuracy of genotype imputation of a low-density SNP array for the Amazon fish Colossoma macropomum
Source: Genet Mol Biol. 2024 Sep 2;47(3):e20230364. doi: 10.1590/1678-4685-GMB-2023-0364 (PMC11445733; doi:10.1590/1678-4685-GMB-2023-0364)
Supplement: Figure S1- [file 1415-4757-GMB-47-03-e20230364-s1.pdf]

Genetics and Molecular Biology
https://doi.org/10.1590/1678-4685-GMB-2023-0364

Supplementary Material to "Accuracy of genotype imputation of a low-density SNP array for the Amazon fish *Colossoma macropomum*"

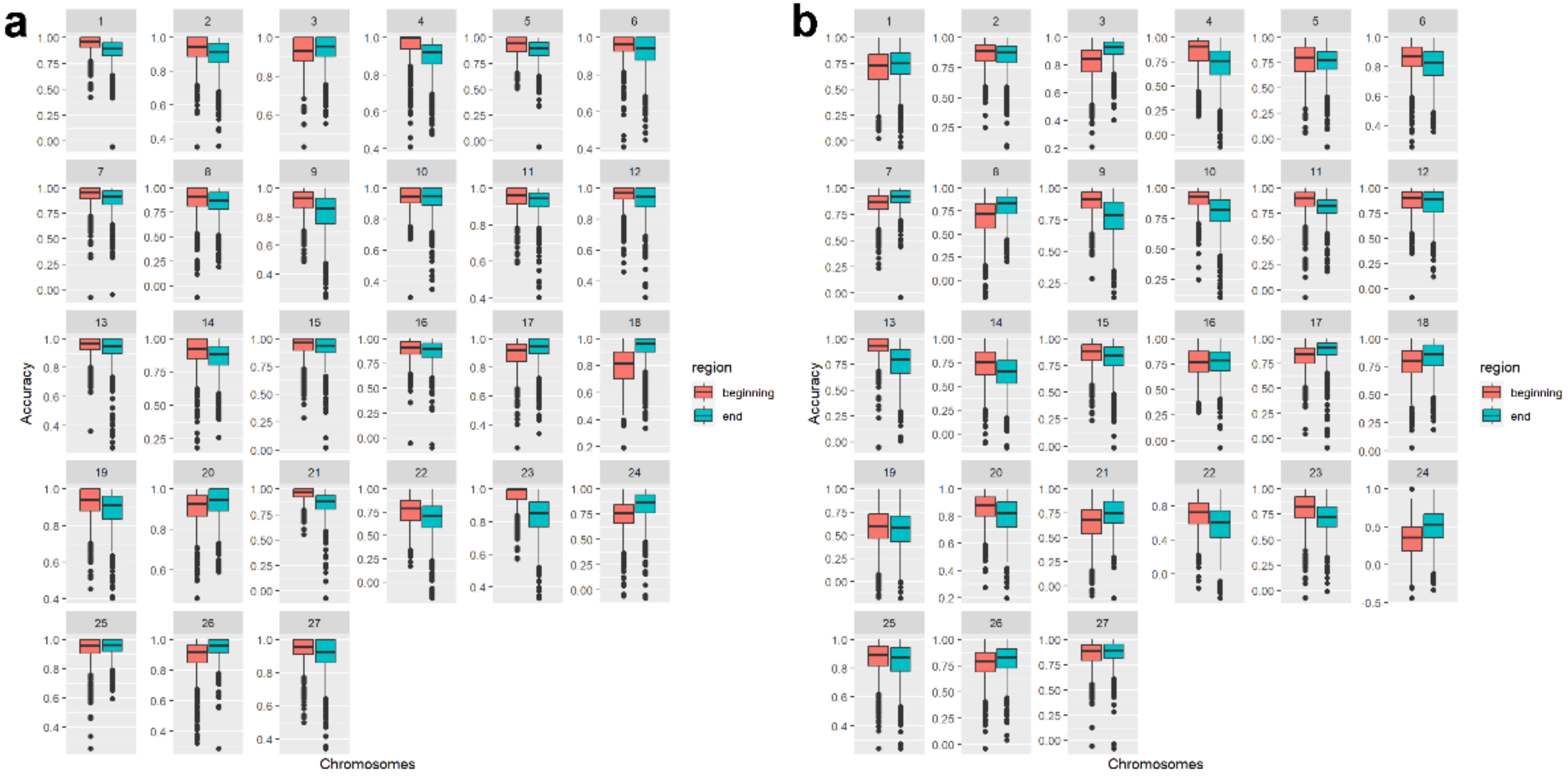

Figure S1 - Mean accuracy (R^2) of imputed extremities (beginning in red and end in blue) of the 27 chromosomes of tambaqui using the 1K (a) and 0.5K (b) low-density subsets. Each chromosome extremity was analyzed by adopting 100 SNP markers.
